# Immunomodulatory Properties of Adipose-Derived Stem Cells Treated with 5-Azacytydine and Resveratrol on Peripheral Blood Mononuclear Cells and Macrophages in Metabolic Syndrome Animals

**DOI:** 10.3390/jcm7110383

**Published:** 2018-10-24

**Authors:** Katarzyna Kornicka, Agnieszka Śmieszek, Agnieszka Sławomira Węgrzyn, Michael Röcken, Krzysztof Marycz

**Affiliations:** 1 Department of Experimental Biology, The Faculty of Biology and Animal Science, University of Environmental and Life Sciences, 50-375 Wroclaw, Poland; kornicka.katarzyna@gmail.com (K.K.); smieszek.agnieszka@gmail.com (A.S.); 2 PORT Polish Center for Technology Development, 54-066 Wrocław, Poland; agnieszka.wegrzyn@eitplus.pl; 3 Faculty of Veterinary Medicine, Equine Clinic-Equine Surgery, Justus-Liebig-University, 35392 Giessen, Germany; Michael.Roecken@vetmed.uni-giessen.de

**Keywords:** adipose-derived stem cells, mesenchymal stem cells, inflammation, autophagy, immunomodulatory effects

## Abstract

Endocrine disorders, including equine metabolic syndrome (EMS), are a serious issue in veterinary medicine and horse breeding. Furthermore, EMS was shown to affect the cytophysiological properties of adipose-derived stem cells, reducing their therapeutic potential. However, it was shown that those cells can be rejuvenated while using a combination of two chemicals: 5-azacytydine (AZA) and resveratrol (RES). In the present study, we decided to evaluate the immunomodulatory properties of AZA/RES-treated adipose-derived stem cells (ASC) isolated from EMS horses (ASC_EMS_). Thus, we co-cultured ASC with peripheral blood mononuclear cells (PBMC) and RAW264.7 macrophages. Most attention was placed on regulatory T lymphocytes (T_REG_), as well as the messenger RNA (mRNA) and protein levels of several cytokines (tumor necrosis factor α (TNF-α), interleukin (IL)-6, IL-10, and IL-1β). Moreover, we also investigated the expression of genes related to auto- and mitophagy in both PBMCs and ASCs. PBMCs were obtained from healthy and EMS-suffering individuals and were co-cultured with ASCs that were isolated from healthy and EMS horses cultured in control conditions and with AZA/RES. We discovered that cells treated with AZA/RES increase the T_REG_ number while co-cultured with PBMCs. Moreover, the co-culture of PBMCs with AZA/RES-treated ASC_EMS_ induced mitophagy in PBMCs. Furthermore, ASC_EMS_ pre-treated with AZA/RES displayed anti-inflammatory properties, as decreased levels of TNF-α, nitric oxide (NO), and IL-6 were observed in those cells in comparison with their untreated counterparts in the co-culture with RAW264.7 macrophages. In summary, we demonstrated that ASC_EMS_ treated with AZA/RES displayed increased anti-inflammatory properties, and was able to regulate and activate the T_REG_-related anti-inflammatory response.

## 1. Introduction

Mesenchymal stem cells (MSCs) are a progenitor stem-cell population isolated from multiple tissues, mainly bone marrow (BMSC) and adipose tissue (ASC). Adipose tissue is the favored source of MSCs because it is accessible in vast amounts and it results in a high yield of isolated cells. ASCs can differentiate into chondrocytes, adipocytes, and osteoblasts, and they display a wide range of immunomodulatory functions, which makes them a valuable tool in cell-based therapies. Furthermore, MSCs are characterized by low immunogenicity and immunoregulatory properties [1]. For these reasons, the application of MSCs in the treatment of autoimmune disorders and tissue damage, and in the induction of allogenic transplant tolerance is fully reasonable and promising [2,3]. MSCs are able to escape immune surveillance and induce allogeneic tissue repair due to the low expression of major histocompatibility complex 1 (MHC-I) antigens, and the lack of major histocompatibility complex 2 (MHC-II), cluster of differentiation 40 (CD40), CD80, and CD86 expressions [4]. However, it was shown that, during in vivo differentiation, the expression of MHC antigens is triggered, although they are not rejected by host organism [5]. The mechanism underlying that phenomenon is still poorly understood. Multiple studies indicated that MSCs regulate T-cell and B-cell functions. They suppress T-cell proliferation and cytokine secretion, and they regulate the T helper 1/T helper 2 (Th1/Th2) cell balance [6,7]. Moreover, they are able to regulate the functions of regulatory T cells (T_REG_) [8], which indicates that MSCs may represent an effective therapeutic strategy for metabolic syndrome treatment.

Equine metabolic syndrome (EMS) is a disorder that is more and more often diagnosed in horses which threatens animal life, as it usually leads to laminitis. EMS is similar to metabolic syndrome in humans (MS) as it is characterized by insulin resistance, visceral obesity, hypertriglyceridemia, and low high-density lipoprotein (HDL) cholesterol levels. Metabolic overload triggers multiple stress reactions in horses suffering from EMS, including oxidative, inflammatory, and organelle stresses in the liver, muscles, and adipose tissue [9]. All of these factors contribute to the pro-inflammatory state of the organism. It was shown that interleukin-1β (IL-1β), interleukin-6 (IL-6), and tumor necrosis factor α (TNF-α) gene expressions were lower or not different in obese, hyperinsulinemic horses compared with animals of normal weight [10]. On the other hand, in EMS horses, no differences were found in the TNF-α, IL-1β, IL-6, plasminogen activator inhibitor 1 (PAI-1), and monocyte chemoattractant protein 1 (MCP-1) expressions in different fat depositions [11]. On the other hand, another study indicated that a positive correlation of TNF and IL-1 expressions, body condition score (BCS), and increased cytokine expression are major risk factors for the development of insulin resistance in horses [12]. Our own previous data showed that IL-6 expression in adipose tissue biopsies that are derived from animals with EMS was enhanced, while TNF-α levels of both groups were comparable. Moreover, EMS horses displayed significantly increased levels of serum IL-6 and TNF-α. Furthermore, histological analysis revealed macrophage infiltration and fibrosis in adipose tissue samples from EMS animals.

The importance of the cytokine profile and its role in the development/maintenance of EMS, due to discrepancies in different research studies, are widely and controversially discussed. However, no data exist regarding the contribution of ASC to inflammation and reduced insulin response in tissues in the course of EMS. As ASCs reside in the pro-inflammatory microenvironment of adipose tissue, they may indeed contribute to or restrict the development of EMS due to their immunoregulatory properties. For this study, we postulated that ASCs from EMS (ASC_EMS_) are not as effective in immunomodulation as ASCs from healthy individuals. Our own previous studies revealed that ASC_EMS_ cells are characterized by decreased viability, senescence, apoptosis, and decreased multipotency capacities in comparison to healthy animals [13,14,15].

For that reason, those cells may also present different behavior in vivo, including a diminished anti-inflammatory response.

Taking into consideration the impairment of ASC_EMS_ cells, in the present study, we decided to rejuvenate those cells in vitro and investigate how this influences their immunomodulatory properties. We treated cells with a combination of 5-azacytydine (AZA) and resveratrol (RES) in order to reverse epigenetic alternations and to reduce the accumulation of oxidative stress factors. Our previous data showed that the application of AZA in human ASCs that were isolated from elderly patients improved their proliferation and reduced apoptosis [16]. Similar results were found for RES, as we showed that polyurethane–polylactide-based material doped with resveratrol decreased the senescence and oxidative stress of ASCs [17]. We hypothesize that autophagy is a mechanism underlying the immunoregulatory properties of ASCs. Autophagic machinery is triggered in stress conditions in order to ensure cell survival. It involves the degradation of cellular components, misfolded proteins, and impaired organelles, delivering valuable building blocks for the synthesis of indispensable biomolecules. Moreover, it plays a crucial role in the regulation of lymphocyte homeostasis and survival, cytokine secretion, and antigen presentation [18]. However, whether autophagy plays a role in regulating ASC-mediated immunomodulation remains elusive.

Based on these observations, in the present study, we decided to investigate whether the treatment of adipose stem cells that were isolated from equine metabolic syndrome individuals with AZA/RES influences their immunogenic properties and immunomodulatory mechanisms in order to provide novel data for the clinical application of rejuvenated cells. To test this hypothesis, we co-cultured ASC_EMS_ and ASC_AZA/RES_ with peripheral blood mononuclear cells (PBMC) and RAW264.7 macrophages.

## 2. Materials and Methods

All of the reagents used in this experiment were purchased from Sigma-Aldrich (Poland), unless indicated otherwise.

### 2.1. Qualification of Horses

Twelve mixed-sex, age-matched (9–14 years; mean ± SD, 11.2 ± 1.7 years) horses, were divided into two groups: a group of individuals suffering from EMS (*n* = 6) and the control group, consisting of animals in good physical condition (*n* = 6), as shown previously [19]. The division was done on the basis of detailed interviews with owners and clinical parameters, such as body weight, body condition score, cresty neck score, combined glucose-insulin test, leptin concentration, and insulin levels. Experimental procedures were approved by the II Local Ethics Committee of Environmental and Life Sciences University (Chelmonskiego 38C, 51-630 Wroclaw, Poland; decision No. 84/2012; extension No. 84/2018).

### 2.2. ASC Isolation and Culture

ASC were isolated from subcutaneous adipose tissue (from the tail base of horses). Tissue specimens were washed with a Hank’s balanced salt solution (HBSS) supplemented with 1% of a penicillin, streptomycin, and amphotericin B (PSA, 10,000 units penicillin, 10 mg streptomycin, and 25 μg amphotericin B per mL) solution and minced into small pieces. Next, samples were digested with collagenase type I (1 mg/mL) for 40 min at 37 °C. Obtained suspension was centrifuged at 1200 × g for 10 min at 23 °C, then pellets were re-suspended in the culture medium and transferred to the culture flasks. Prior experiments, ASC from experimental group were treated for 24 h with combination of AZA (0.5 µM) and RES (0.05 µM) diluted in water.

Cultures were maintained under constant conditions at 37 °C, 95% humidity, and 5% CO_2_. The cells were cultured in Dulbecco’s modified Eagle’s medium (DMEM) low glucose supplemented with 10% of fetal bovine serum (FBS) and 1% PSA solution. Medium was changed every two days. After reaching 80–90% of confluence, the cells were passaged using a trypsin solution (TrypLE Express, Life Technologies, Carlsbad, CA, USA). At passage 3, MSCs were collected and cellular phenotype confirmed by the expression of markers CD_44_, CD_90_, and CD_45_, as well as by their capacity for tri-lineage differentiation, as previously shown [19].

### 2.3. Extraction and Culture of Peripheral Blood Mononuclear Cells (PBMC)

Fresh blood was collected from horses into syringes filled with heparin. PBMCs were isolated using Ficoll Histopaque^®^-1077 density gradient centrifugation at 400× *g*, at 4 °C for 20 min. Cells from buffy coat layer were collected and washed three times with HBSS. Next, cells were re-suspended in one complete culture medium (RPMI 1640 medium supplemented with 10% FBS, 1% PSA) and transferred to culture dish.

### 2.4. Flow Cytometry Analysis

PBMCs isolated from horses were stained against CD_4_ and CD_25_. Briefly, cells were incubated with mouse anti horse CD_4_ (MCA1078GA, 1:200; Abd Serotec, Hercules, CA, USA) in 4 °C for 30 min. Next, cells were washed three times and incubated with secondary antibody (A-31571, 1:1000; Alexa Fluor Plus 647 Thermo Fisher Scientific, Carlsbad, CA, USA). Following this, cells were stained with mouse anti-human CD_25_ conjugated with FITC (MA1-35144, 1:200; Thermo Fisher Scientific, Carlsbad, CA, USA). The same procedure was applied to PBMC after co-culture with ASCs. After last staining, cells were washed cells and resuspended in PBS. The measurements were performed using BD LSR Fortessa (Becton-Dickinson and Company, Franklin Lakes, NJ, USA). At least 20,000 cells were analysed. The data analysis was performed using FlowJo software (TreeStar Inc., Ashland, OR, USA). The first gate include lymphocytes, which were than gated and analysed for CD_4_ expression. Further the CD_4_^+^ population was analysed for the expression of CD_25_. Additionally, within the population of CD_4_^+^/CD_25_^+^ cells, we gated the population with the distinct brighter fluorescence signal i.e., CD25^high^ cells.

#### 2.4.1. ASC-PBMC Co-Culture

ASCs were seeded on 24-well plates at the density of 4 × 10^4^ 24 h prior co-culture with PBMCs. Cells were cultured in DMEM-LG supplemented with 10% FBS and 1% PSA. The isolated PBMCs (5 × 10^5^ cells/well) were seeded onto 8 µm transwell and co-cultured with pre-seeded ASC. After 24 h the supernatant was collected for further analysis and cells were lysed in TRI Reagent.

#### 2.4.2. ASC-RAW264.7 Co-Culture

RAW 264.7 were counted and seeded at the density of 1 × 10^6^ cells/mL per well of 24-well plate in a volume of 500 μL/well. After 18 h, non-adherent cells were removed by a gentle wash with warm HBSS. Next, LPS was added to the culture media at a concentration of 1 μg/mL. At the same time, ASC in the amount of 4 × 10^4^ was added to culture wells. After 24 h of co-culture, media were collected for an analysis of the macrophages’ secretory activity, and the cells were lysed by adding TRI Reagent.

### 2.5. Nitric Oxide (NO) and Superoxide Dismutase (SOD) Levels Analysis

SOD activity analysis was conducted using a SOD Assay Kit, and the level of nitric oxide (NO) was assessed with a Griess reagent kit (Life Technologies, Carlsbad, CA, USA). All of the procedures were performed in accordance to manufacturer’s instructions.

### 2.6. Real-Time Reverse Transcription Polymerase Chain Reaction (qRT-PCR)

Total RNA was isolated from cells according to a single-step method that was described previously by Chomczynski and Sacchi [20] and Reverse Transcription Polymerase Chain Reaction (RT-PCR) was performed as described elsewhere [14]. RNA quality and quantity were determined with spectrophotometry (Epoch, BioTek, Winooski, VT, USA). 150 ng of total RNA was used for genomic DNA digestion and cDNA synthesis. Digestion of genomic DNA was performed using DNase I, RNAase-free (Life Technologies) while complementary DNA (cDNA) synthesis were performed using RevertAid RT Reverse Transcription Kit (Life Technologies). For each reaction, 150 ng of total RNA was used. Genomic DNA digestion and cDNA synthesis were performed using a T100 Thermal Cycler (Bio-Rad, Hercules, CA, USA). The qRT-PCR reactions were carried out using a SensiFAST SYBR Green Kit (Bioline, London, UK). Primer concentration equalled 0.5 μM. Sequences of the primers that were used in reactions are listed in Table 1. All qRT-PCR reactions were conducted with CFX Connect™ Real-Time PCR Detection System (Bio-Rad). Relative gene expression analysis (Qn) were evaluated in relation to the GAPDH as a housekeeping gene using ΔΔCt method. Moreover, the ratio of MFN/FIS expression was determined by dividing ΔΔCt of genes.

### 2.7. ELISA Tests

Using ELISA amount of TNF-α (Mouse TNF-α Quantikine ELISA Kit, R&D Systems, Minneapolis, MN, USA), IL-6 (My Biosource, San Diego, CA, USA), IL-10 (Thermo Fisher Scientific, Carlsbad, CA, USA), and IL-β (R&D Systems, Minneapolis, MN, USA) was evaluated. Procedures and calculations were carried out in accordance with the manufacturer’s instruction. The absorbance was measured with a 96-well microplate reader (Epoch, BioTek) at 450 nm.

### 2.8. Statistical Analysis

Differences between experimental groups were estimated using ANOVA one way analysis of variance. Statistical analysis was conducted with GraphPad Prism 5 Software (La Jolla, CA, USA). Differences with probability of *p* < 0.05 were considered to be significant. Statistical significance was labelled with hash (#) when comparing to control group (CTRL) (ASC_CTRL_/PMBM_CTRL_) and asterisk (*) when comparing to the EMS group (ASC_EMS_/PBMC_EMS_).

## 3. Results

### 3.1. Flow Cytometry Analysis of PBMCs

**Figure 1:**
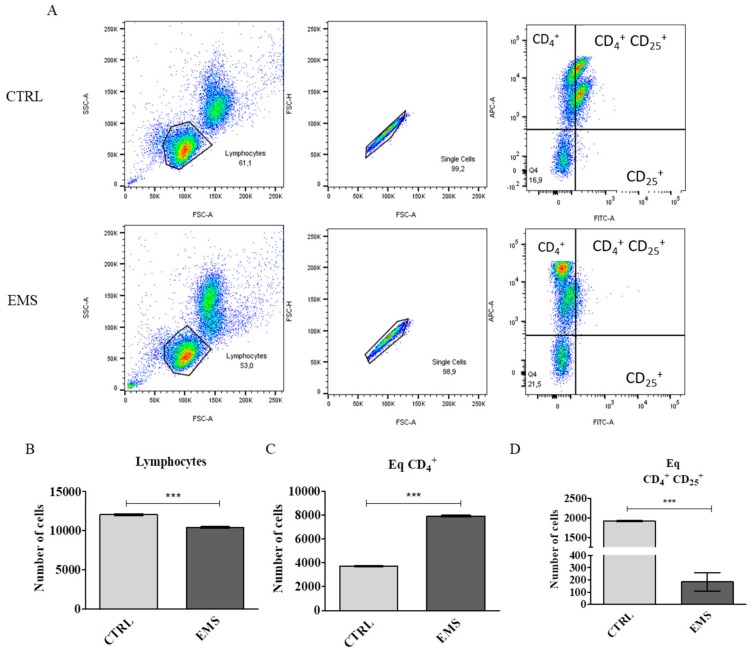
Flow cytometry analysis of CD_4_^+^ CD_25_^+^ regulatory T cells. Representative graphs showing dot plots from flow cytometry analysis (**A**). Total number of lymphocytes was significantly decreased in equine metabolic syndrome (EMS) horses (**B**), while number of CD_4_^+^ cells increased (**C**). Furthermore, EMS horses were characterized by decreased number of T_REG_ in circulating blood (**D**). Results expressed as mean ± S.D. *** *p* < 0.001.

In order to investigate changes in blood cells number, peripheral mononuclear cells (PBMCs) were isolated from fresh blood using histopaque gradient. Next, PBMCs from control and EMS individuals were subjected to flow cytometry analysis. Representative graphs are shown in Figure 1A. The total number of lymphocytes was significantly lower in EMS horses (Figure 1B, *p* < 0.001) while CD_4_^+^ cells increased (Figure 1C, *p* < 0.001). On the other hand, percentage of CD_4_^+^ CD_25_^+^ regulatory cells was downregulated in EMS horses in comparison to healthy individuals (Figure 1D, *p* < 0.001).

**Figure 2:**
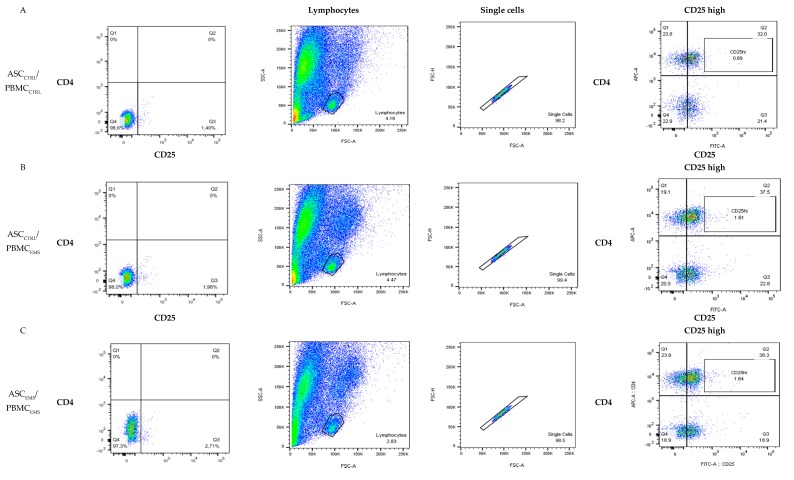

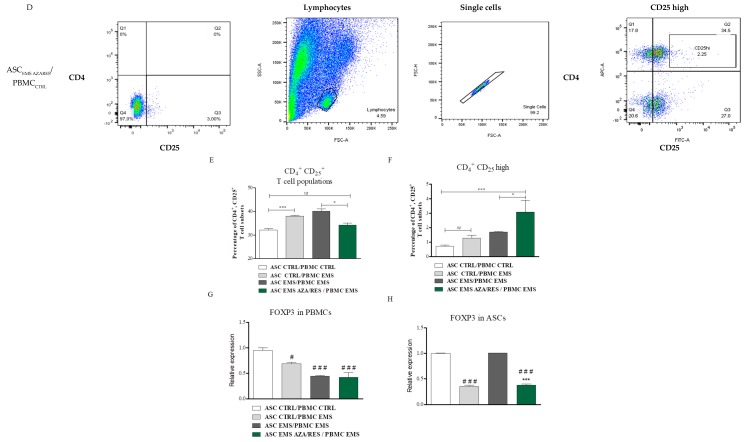
Activation of CD_4_^+^ CD_25_^+^ regulatory T cells in co-culture with adipose-derived stem cells (ASC). Graphs are showing representative results of flow cytometry analysis (**A**–**D**). Peripheral blood mononuclear cells (PBMC) after co-culture with ASC were subjected for analysis of CD_4_^+^ CD_25_^+^ T cells population (**E**) and CD_4_^+^ CD_25_ high T cells (**F**) using flow cytometry. Furthermore, using RT-PCR expression of FOXP3 was investigated both in PBMC (**G**) and ASC (**H**). Statistical significance was labelled with hash (#) when comparing to control group (ASC_CTRL_/PMBM_CTRL_) and asterisk (*) when comparing to EMS group (ASC_EMS_/PBMC_EMS_). Results expressed as mean ± S.D. #, *: *p* < 0.05; ###, ***: *p* < 0.001.

Next, PBMCs were co-cultured with ASC isolated from healthy and EMS individuals for 24 h. Using flow cytometry number of T_REGS_ after co-culture was examined. Representative graphs are shown on Figure 2A–D. Quantification of obtained data revealed that co-culture of ASC_CTRL_ with PBMC_EMS_, ASC_EMS_ with PBMC_EMS_, and ASC_EMS AZA/RES_ with PBMC_EMS_ increased the number of CD_4_^+^ CD_25_^+^ T cell population (Figure 2E) in comparison to ASC_CTRL_ with PBMC_CTRL_ co-culture. Furthermore, ASC_EMS AZA/RES_ co-cultured with PBMC_EMS_ enhanced activation of T_REG_ as significantly increased number of CD_4_^+^ CD_25_^+^ high cells number was noted in that group (Figure 2F). Interestingly, the expression of FOXP3 in PBMCs after co-culture was downregulated in all investigated groups in comparison to ASC_CTRL_ cultured with PBMC_CTRL_ (Figure 2G). What is more, we have also examined expression of FOXP3 in ASC. Analysis revealed decreased gene expression in ASC_CTRL_ cultured with ASC_EMS_ and ASC_EMS AZA/RES_ co-cultured with PBMC_EMS_ (Figure 2H).

### 3.2. Gene Expression After ASC-PBMC Co-Culture

**Figure 3:**
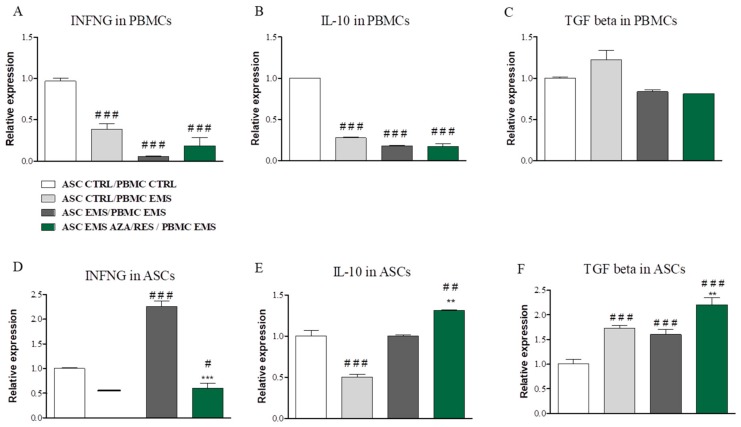
Gene expression profile in PBMCs and ASCs after co-culture. Using RT-PCR expression of INFNG (**A**), IL-10 (**B**), and tumor growth factor (TGF) beta (**C**) was examined in PBMCs after co-culture with ASCs. The mRNA levels of the same genes-INFNG (**D**), IL-10 (**E**), and TGF beta (**F**) was also investigated in ASCs. Statistical significance was labelled with hash (#) when comparing to control group (ASC_CTRL_/PMBM_CTRL_) and asterisk (*) when comparing to the EMS group (ASC_EMS_/PBMC_EMS_). Results expressed as mean ± S.D. #: *p* < 0.05; ##, ** *p* < 0.01; ###, *** *p* < 0.001.

After co-culture, both ASC and PBMC were lysed in TRIReagent in order to RNA isolation. Next, specimens were subjected for RT-PCR analysis. INFNG expression in PBMCs was downregulated in ASC_CTRL_/PBMC_EMS_ (*p* < 0.001), ASC_EMS_/PBMC_EMS_ (*p* < 0.001), and in ASC_EMS AZA/RES_/PBMC_EMS_ (*p* < 0.001) in comparison to the control group (Figure 3A). A similar trend was observed in the expression of IL-10 (Figure 3B), while no differences were observed in the expression of TGF beta in investigated PBMCs (Figure 3C). For ASC, no expression of INFNG was found in ASC_CTRL_/PBMC_EMS_, while its expression was significantly up-regulated in ASC_EMS_ co-cultured with PBMC_EMS_ (*p* < 0.001) (Figure 3D). Furthermore, INFNG expression was downregulated (*p* < 0.05) in ASC_EMS AZA/RES_ co-cultured with PBMC_EMS_ (Figure 3D) in comparison to control group. IL-10 expression was downregulated in ASC_EMS_ co-cultured with PBMC_EMS_ (Figure 3E, *p* < 0.001), while it was increased in ASC_EMS AZA/RES_ co-cultured with PBMC_EMS_ (Figure 3E, *p* < 0.01). TGF beta expression (Figure 3F) was increased (*p* < 0.001) in all the investigated groups in comparison to control group (ASC_CTRL_/PBMC_CTRL_).

### 3.3. Cytokines, NO, and SOD Amount after ASC-PBMC Co-Culture

**Figure 4:**
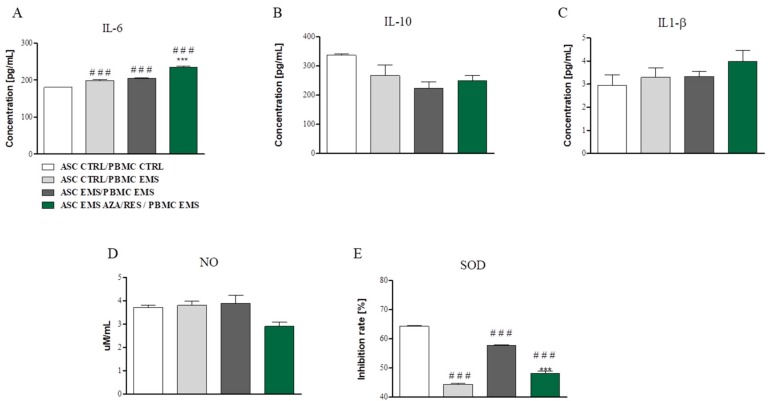
Cytokines, nitric oxide (NO), and superoxide dismutase SOD amounts evaluated after co-culture of ASCs with PBMCs. Culture media after co-culture was collected and subjected for analysis. Using ELISA (**A**) amount of IL-6 (**A**), IL-10 (**B**) and IL-1β (**C**) was evaluated. Using spectrophotometric assays NO (**D**) and SOD (**E**) levels were investigated. Statistical significance was labelled with hash (#) when comparing to control group (ASC_CTRL_/PMBM_CTRL_) and asterisk (*) when comparing to the EMS group (ASC_EMS_/PBMC_EMS_). Results expressed as mean ± S.D. ###, *** *p* < 0.001.

In order to perform the assays culture media after co-culture was collected and analysed. Using ELISA, IL-6, IL-10, and IL-1β secretion was examined. Obtained data indicated an increased amount of IL-6 (Figure 4A) in all experimental groups in comparison to control (*p* < 0.001). Interestingly, no differences in the levels of, IL-10 (Figure 4B) neither IL-1β (Figure 4C) was observed. Furthermore, using Griess reagent amount of NO in culture media was established (Figure 4D), however no statistically significant differences were found between investigated groups. SOD activity was ameliorated in all experimental groups when compared to the control one (Figure 4E, *p* < 0.001).

### 3.4. Evaluation of Autophagy-Related Genes after ASC-PBMC Co-Culture

**Figure 5:**
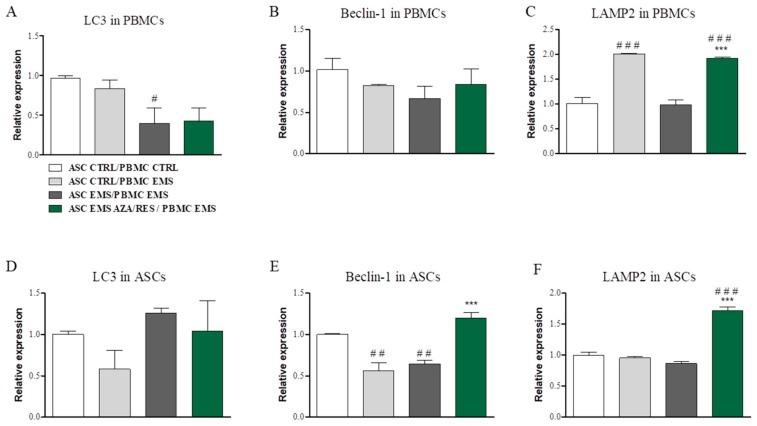
Evaluation of autophagic genes after co-culture of ASCs with PBMCs. Using RT-PCR expression of LC3 (**A**), Beclin-1 (**B**), and LAMP-2 in PBMCs were analysed (**C**). Similarly mRNA levels of LC3 (**D**), Beclin-1 (**E**) and LAMP-2 (**F**) were investigated in ASC. Statistical significance was labelled with hash (#) when comparing to control group (ASC_CTRL_/PMBM_CTRL_) and asterisk (*) when comparing to EMS group (ASC_EMS_/PBMC_EMS_). Results expressed as mean ± S.D. # *p* < 0.05; ## *p* < 0.01; ###, *** *p* < 0.001.

In order to evaluate the levels of autophagy, expression of LC3, Beclin-1 and LAMP2 was evaluated in both ASC and PBMC after co-culture for 24 h. LC3 expression was significantly downregulated in PMBC_EMS_ after co-culture with ASC_EMS_ (Figure 5A, *p* < 0.05), while no differences were noted in the expression of Beclin-1 in investigated PBMCs (Figure 5B). LAMP2 expression was significantly increased in ASC_CTRL_/PBMC_EMS_ and ASC_EMS AZA/RES_/PBMC_EMS_ groups (Figure 5C, *p* < 0.001). In ASC, no differences were found in the expression of LC3 (Figure 5D). Beclin-1 mRNA was downregulated (Figure 5E) in ASC_CTRL_ cultured with PBMC_EMS_ and in ASC_EMS_ co-cultured with PBM_CEMS_ (*p* < 0.001). Interestingly, ASC_EMS AZA/RES_ co-cultured with PBMC_EMS_ were characterized by increased Beclin-1 expression (*p* < 0.001) in comparison to ASC_EMS_ that are co-cultured with PBMC_EMS_. Similar trend was observed in the expression of LAMP2 (Figure 5F) in ASC_EMS AZA/RES_.

### 3.5. Evaluation of Mitophagy-Related Genes after Co ASC-PBMC Co-Culture

**Figure 6:**
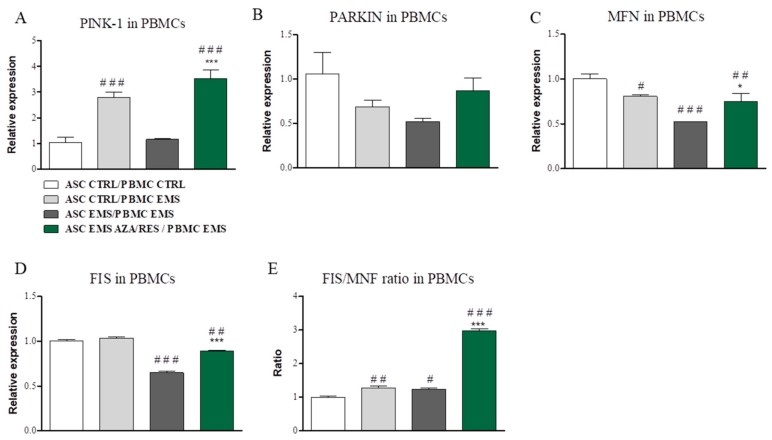

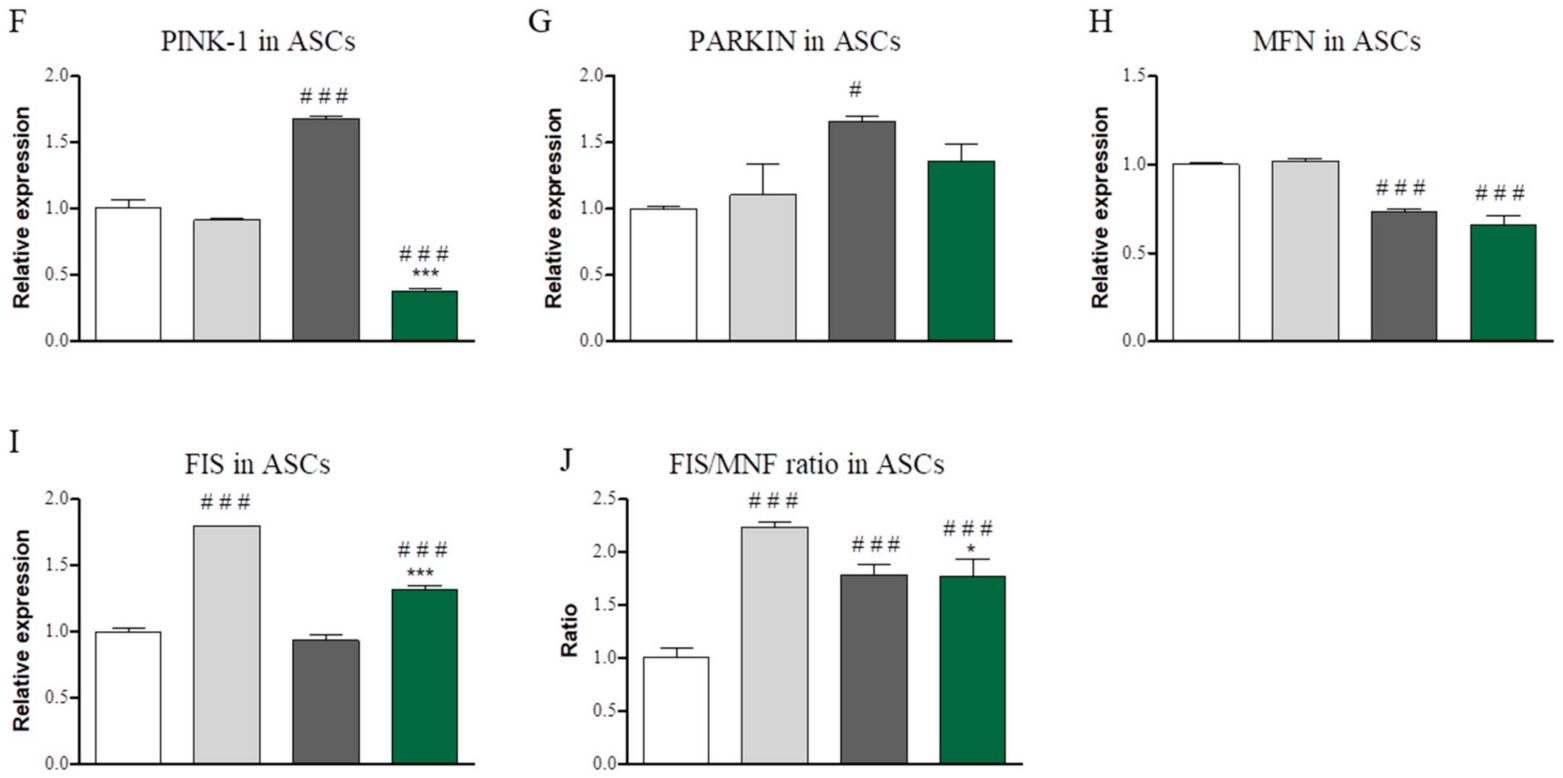
Evaluation of mitophagy-related genes after co-culture of ASCs with PBMCs. After co-culture expression of PINK-1 (**A**), PARKIN (**B**), MFN (**C**), and FIS (**D**) in PBMCs was evaluated using RT-PCR. Furthermore, the ratio of FIS/MNF (**E**) was calculated based on relative expression of both genes. The same genes: PINK-1 (**F**), PARKIN (**G**), MFN (H), and FIS (**I**) mRNA levels were established for ASCs. Similarly, ratio of FIS/MNF expression was calculated (**J**). Statistical significance was labelled with hash (#) when comparing to control group (ASC_CTRL_/PMBM_CTRL_) and asterisk (*) when comparing to the EMS group (ASC_EMS_/PBMC_EMS_). Results expressed as mean ± S.D. #, * *p* < 0.05; ## *p* < 0.01; ###, *** *p* < 0.001.

In order to evaluate mitophagy, cells were subjected for RT-PCR analysis. Expression of following genes was examined: PINK-1, PARKIN, MFN, and FIS. PINK-1 expression was upregulated (*p* < 0.001) in PBMC_EMS_ co-cultured with ASC_EMS_ and ASC_EMS AZA/RES_ (Figure 6A). No statistically significant differences were observed in the expression of PARKIN between the investigated groups (Figure 6B). MFN expression in PBMC was downregulated in experimental groups in comparison to control (ASC_CTR_/PBMC_CTRL_) (Figure 6C). FIS expression was downregulated in PBMC co-cultured with ASC_EMS_ and ASC_EMS AZA/RES_ (Figure 6D, *p* < 0.01 and *p* < 0.001, respectively). The FIS/MNF ratio was increased in all investigated groups however most significantly in PBMCs co-cultured with ASC_EMS AZA/RES_ (Figure 6E, *p* < 0.001). In ASC, PINK-1 expression was upregulated in ASC_EMS_ cells co-cultured with PBMC_EMS_ (*p* < 0.001) while decreased in ASC_EMS AZA/RES_ (*p* < 0.001) (Figure 6F). PARKIN expression was only up-regulated in ASC_EMS_ co-cultured with PBMC_EMS_ (Figure 6G, *p* < 0.05). MFN mRNA levels were decreased in ASC_EMS_ co-cultured with PBMC_EMS_ and in ASC_EMS AZA/RES_ co-cultured with PBMC_EMS_ (Figure 6H, *p* < 0.001). FIS expression was significantly increased in ASCCTRL that was co-cultured with PBMCEMS and in ASC_EMS AZA/RES_ co-cultured with PBMC_EMS_ (Figure 6I, *p* < 0.001). FIS/MFN ratio was significantly increased in all experimental groups in comparison to control (Figure 6J, *p* < 0.001).

### 3.6. Co-Culture of ASC with RAW264.7 Macrophages

**Figure 7:**
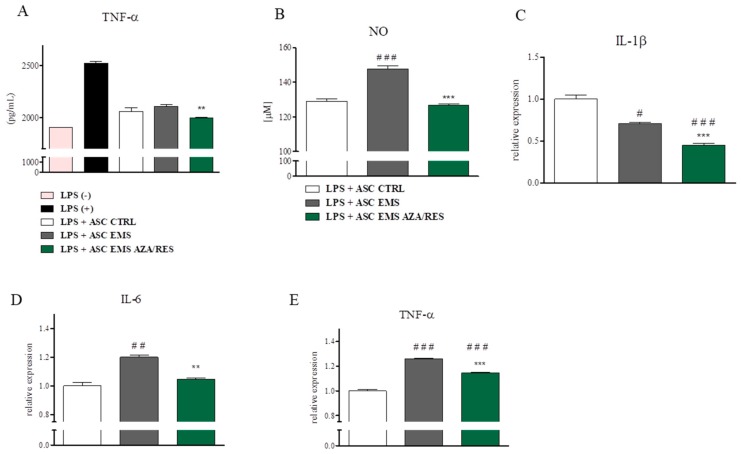
Anti-inflammatory properties of ASC s cultured with RAW 264.7 macrophages. ASCs were co-cultured with LPS-treated macrophages in order to evaluate their immunomodulatory properties. After co-culture culture media was collected and subjected for TNF-α (**A**) analysis with ELISA test. Using Griess reagent amount of NO was examined (**B**). RT-PCR was performed to investigate expression of IL-1β (**C**), IL-6 (**D**), and TNF-α (**E**). Statistical significance was labelled with hash (#) when comparing to control group (LPS+ASC_CTRL_) and asterisk (*) when comparing to EMS group (LPS+ASC_EMS_). Results expressed as mean ± S.D. #, *p* < 0.05; ##, ** *p* < 0.01; ###, *** *p* < 0.001.

In order to evaluate the immunomodulatory properties of ASC they were incubated with LPS-stimulated RAW264.7 macrophages for 24 h. Next, cells and culture media were subjected for further analysis. ELISA for TNF-α revealed its decreased levels in ASC_EMS AZA/RES_ group in comparison to untreated ASC_EMS_ (Figure 7A, *p* < 0.01). NO amount was increased in ASC_EMS_ (*p* < 0.001, Figure 7B), however, in the ASC_EMS AZA/RES_ group its levels were decreased (*p* < 0.001). IL-1β expression (Figure 7C) was downregulated in both ASC_EMS_ and ASC_EMS AZA/RES_ (*p* < 0.05 and *p* < 0.001 respectively). IL-6 expression (Figure 7D) was upregulated in ASC_EMS_ in comparison to ASC_CTRL_ (*p* < 0.01) while downregulated (*p* < 0.01) in ASC_EMS AZA/RES_ when comparing to untreated cells. TNF-α mRNA level (Figure 7E) was increased (*p* < 0.001) in both ASC_EMS_ and ASC_EMS AZA/RES_ while comparing to ASC_CTRL._ However its expression was decreased in ASC_EMS AZA/RES_ in comparison to ASC_EMS_ (*p* < 0.001).

### 3.7. Auto- and Mitophagy after ASC-RAW264.7 Co-Culture

**Figure 8:**
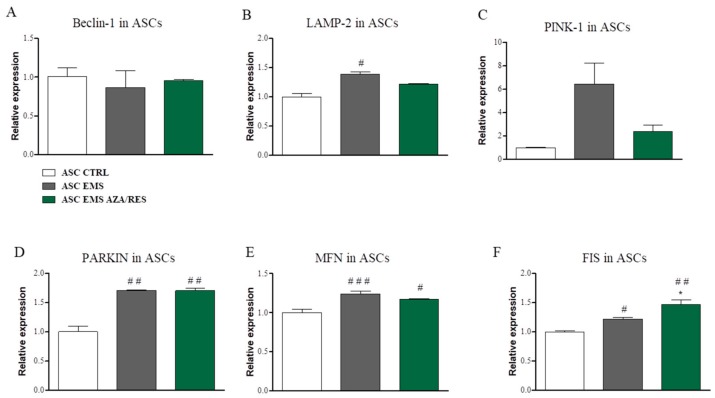
Auto- and mitophagy in ASCs co-cultured with RAW264.7 macrophages. After co-culture, ASC were collected and subjected for RT-PCR. Expression of Beclin-1 (**A**), LAMP-2 (**B**), PINK-1 (**C**), PARKIN (**D**), MFN (**E**), and FIS (**F**) was evaluated. Statistical significance was labelled with hash (#) when comparing to control group (ASC_CTRL_) and asterisk (*) when comparing to EMS group (ASC_EMS_). Results expressed as mean ± S.D. #, * *p* < 0.05; ## *p* < 0.01; ### *p* < 0.001.

In order to evaluate levels of autophagy and mitophagy in ASC after co-culture with macrophages following genes expression was examined with RT-PCR: Beclin-1, LAMP-2, PINK-1, PARKIN, MFN, and FIS. No statistically significant differences were noted in the expression of Beclin-1 between investigated groups (Figure 8A). LAMP2 mRNA levels were increased in ASC_EMS_ in comparison to control cells (Figure 8B, *p* < 0.05). Furthermore, no significant differences were found in the expression of PINK-1 between investigated groups (Figure 8C). PARKIN expression was significantly upregulated in ASC_EMS_ and ASC_EMS AZA/RES_ while comparing to ASC_CTRL_ (Figure 8D, *p* < 0.01). MFN expression (Figure 8E) was increased in ASC_EMS_ (*p* < 0.001) and ASC_EMS AZA/RES_ (*p* < 0.05) in comparison to control cells. Similar trend was observed regarding FIS expression (Figure 8F) as it was increased in ASC_EMS_ (*p* < 0.05) and ASC_EMS AZA/RES_ (*p* < 0.01).

## 4. Discussion

In our previous study, we reported that the treatment of ASC_EMS_ with AZA/RES reversed their aged phenotype and rejuvenated them, so that their physiological properties were comparable to those observed in ASC_CTRL_. In the present study, we have investigated immunomodulatory properties of these cells in co-culture with PBMCs and RAW264.7 macrophages. We have found for the first time that ASC_EMS_ and ASC_EMS_ that are treated with AZA/RES induced and increased Treg activity and caused dynamic alternations in the cytokine expression pattern.

Systemic obesity and metabolic syndrome-related abnormalities in the peripheral blood T-cell compartment are not well characterized. In the current study, we have evaluated for the first time the peripheral blood T-cell compartment in healthy and EMS horses. A large body of evidence indicates a pathological role of CD_4_^+^ T cells in obesity and insulin resistance. We have discovered an increased number of CD_4_^+^ cells in EMS individuals. This is consistent with the study that was conducted by van der Weerd et al. [21], who discovered that CD_4_^+^ T-cell number correlated positively with fasting insulin levels in obese patients. A study performed by Shirakawa et al. [22] demonstrated elevated levels of activated CD_4_^+^ T cells in the visceral adipose tissue of obese mice. It has been suggested that obesity triggers major histocompatibility complex (MHC) class II expression on adipocytes and activates CD_4_^+^ T cells to initiate inflammation in adipose tissue. This is in line with our previous data, because we have shown increased inflammation of adipose tissue in EMS individuals [23]. Tregs belong to a small subset of T lymphocytes, constituting about 5–20% of CD_4_^+^. However, they are believed to be important players in preventing inflammatory responses and tissue damage [24]. Furthermore, they are characterized by high-level expression of a forkhead/winged-helix transcription factor, FOXP3. In the present study, we have observed significantly decreased Treg count in the blood of EMS horses, which may be the cause of prolonged inflammation and insulin resistance in these subjects. It was shown that Treg cells could suppress Th1, Th2, and Th17 response in order to enhance insulin resistance. Furthermore, the number of Tregs was also decreased in patients with type 2 diabetes and metabolic syndrome [25,26], while their high number was recorded in lean mice [27]. Thus, Tregs may become a valuable therapeutic tool in EMS by limiting proinflammatory milieu and increasing insulin sensitivity. Interestingly, we observed that ASC_EMS_ treated with AZA/RES increased the number of Tregs in co-culture with PBMCs. Hence, it is likely that after the application of these cells to EMS individuals, the number of circulating Tregs in their blood may increase. Interestingly, mRNA levels of FOXP3 in ASC_EMS_ treated with AZA/RES were decreased. However, it was shown that FOXP3-decreased MSCs retained their immunosuppressive properties [28].

It has been shown that cytokines produced by immune cells modulate MSC fate and function leading to the release of immunomodulatory and growth factors [29,30]. After PBMC co-culture with ASCs, we have investigated mRNA levels of selected cytokines. Interestingly, INFNG expression in PBMCs from EMS horses was significantly decreased in comparison to the control group. The precise mechanisms of these phenomena remain unclear. However, a decreased response of INFNG that is caused by PBMC was also observed in patients with Graves’ disease, Hashimoto’s thyroiditis, and rheumatoid arthritis [31]. We hypothesize that defects in INFNG synthesis may result from changes in ASC_EMS_ response to different stimuli and might be related to disease activity. Interestingly, both IL-10 and TGF beta levels were elevated in ASC_EMS_ treated with AZA/RES. It was shown that the increased production of IL-10 by MSCs inhibited Th17 differentiation, an important proinflammatory CD4+ T cell subtype that is associated with type 2 diabetes [32]. On the other hand, it was demonstrated that TGF beta is an essential factor by which MSCs activate Tregs [33]. It was observed in the co-culture of ASC_EMS_ pre-treated with AZA/RES with PBMCs that the increased TGF beta production by ASC_EMS_ treated with AZA/RES correlated with the increased number of Tregs. Total level of IL-6 measured in the supernatant by ELISA was increased in both ASC_EMS_ and ASC_EMS_ treated with AZA/RES. This may be correlated with the fact that elevated serum levels of IL-6 are recognized as an early and representative marker in metabolic syndrome pathogenesis [34].

Autophagy is recognized as a process that allows for the degradation of cellular components by the lysosomes. Previous studies have indicated that autophagy plays a role in lipotoxicity-induced apoptosis and inflammation [35]. However, its function in inflammatory response of PBMCs co-cultured with ASCs is not fully understood. We observed that LAMP-2 expression was significantly elevated in PBMCs co-cultured with ASC_EMS_ after AZA/RES pre-treatment. LAMP-2 is a protein that is necessary for the maturation of autophagosomes and cargo removal. This indicates that ASC_EMS_ after AZA/RES treatment induces autophagy in PBMCs. Interestingly, LAMP-2 down-regulation in PBMCs cultured with ASC_EMS_ may be a novel mechanism that connects obesity and inflammation in EMS horses. It was shown that reduced autophagy in PBMCs in type 2 diabetes patients was correlated with an enhanced inflammation [36]. Therefore, it is tempting to speculate that rejuvenated ASCs with AZA/RES will affect the physiological properties of PBMCs after clinical application in EMS individuals by triggering autophagy, and, in consequence, diminishing the inflammatory state of the animal body.

Mitophagy involves the selective engulfment of impaired mitochondria into autophagosomes and it serves as a quantity and quality control mechanism [37]. Mitophagy is controlled by different genes, including PTEN-induced putative kinase 1 (PINK1), PARKIN, microtubule-associated protein light chain 3 (LC3), lysosome-associated membrane protein-2 (LAMP-2), mitofusin2 (MFN2), and mitochondrial fission protein (FIS) [38]. Our study is the first report demonstrating that co-culture of PBMCs with AZA/RES-treated ASC_EMS_ induces mitophagy in PBMCs. Thus, it reverses the changes observed in PBMCs from type 2 diabetic patients, who are characterized by down-regulation of MFN2, PINK1, PARKIN, and LAMP-2 genes at the mRNA and protein level. The observed increased mitophagy enables elimination of dysfunctional mitochondria, thereby preventing their accumulation in cells and exacerbation of mitochondrial oxidative stress induced by hyperglycemia. Mitochondrial fission results in the formation of small individual mitochondria, while interconnected networks of these organelles are generated during fusion. In the present study, we observed the up-regulation of fission in PBCMs and co-cultured ASC_EMS_ treated with AZA/RES, which indicated increased mitophagy. Studies have demonstrated that the loss of mitophagy can lead to the accumulation of ROS and induce immune signaling pathways leading to the release of pro-inflammatory cytokines [39]. Thus, the up-regulation of mitophagy observed in the experimental group may contribute to the shift towards anti-inflammatory phenotype of these cells. Mitophagy may be a crucial factor controlling inflammatory response in immune cells.

We co-cultured AZA/RES-treated ASCs with LPS-stimulated RAW264.7 macrophages to further investigate their immunomodulatory properties. ASCs isolated from EMS individuals were characterized by increased expression of NO, IL-6, and TNF-α. On the other hand, ASC_EMS_ pre-treated with AZA/RES displayed anti-inflammatory properties, as decreased TNF-α, NO, and IL-6 levels were observed in these cells in comparison to their untreated counterparts. This phenomenon is in good agreement with the results of Wang et al. [40], who observed that the combined treatment with mouse bone marrow MSCs and resveratrol suppressed proinflammatory cytokines (IFN-γ, TNF-α) and increased anti-inflammatory cytokines (IL-4, IL-10) secretion in experimental autoimmune encephalomyelitis. Anti-inflammatory properties of resveratrol have been demonstrated in pancreatitis [41], arthritis [42], and experimental colitis [43]. Wang et al. discovered that RES prevented the suppression of Treg production, oxidative stress, and inflammation of mice that are prone or resistant to high fat diet-induced obesity [44]. Moreover, similarly to PBMCs-ASCs co-culture, ASC_EMS_ pre-treated with AZA/RES were characterized by increased mitophagy when co-cultured with RAW264.7 macrophages. Thus, we hypothesize that these mechanisms are mainly responsible for immune response modulation in ASCs treated with AZA/RES.

## 5. Conclusions

In summary, our study demonstrated that ASC_EMS_ treated with a combination of AZA/RES displayed enhanced anti-inflammatory properties and were able to regulate and activate Tregs-related anti-inflammatory response. These observations correlate with the occurrence of mitophagy and mitochondrial fission, both in ASCs and immune cells. We postulate that the anti-inflammatory phenotype of these cells requires the modulation of mitochondrial dynamics. Our results provide a potential interventional strategy for managing inflammation in EMS horses by the application of AZA/RES-rejuvenated ASCs.

## Figures and Tables

**Table 1:** Sequences of primers used in Reverse Transcription Polymerase Chain Reaction (RT-PCR).

Gene	Primer	Sequence 5′-3′	Amplicon Length (bp)	Accession no.
*INFNG*	F:	CACCAGCAAGCTGGAAGACT	163	NM_001081949.1
	R:	CCGGCCTCGAAATGGATTCT		
*FOXP3*	F:	AGATGCTGGCCGAGGTCAAC	149	XM_023633195.1
	R:	TGCGGAACTCGAACTCATCC		
*IL-10*	F:	ATAAGAGCAAGGCAGTGGAGC	77	NM_001082490.1
	R:	ACTCATGGCTTTGTAGACACC		
*TGF beta*	F:	ATTCCTGGCGCTACCTCAGT	197	NM_001081849.1
	R:	GCTGGAACTGAACCCGTTGAT		
*LC3*	F:	TTCTGAGACACAGTCGGAGC	128	XM_001493613.6
	R:	CTTTGTTCGAAGGTGTGGCG		
*Beclin–1*	F:	GATGCGTTATGCCCAGATGC	233	XM_014833759.1
	R:	AACGGCAGCTCCTCTGAAAT		
*LAMP-2*	F:	GCACCCCTGGGAAGTTCTTA	147	XM_014831347.1
	R:	ATCCAGCGAACACTCTTGGG		
*PINK-1*	F:	GCACAATGAGCCAGGAGCTA	298	XM_014737247.1
	R:	GGGGTATTCACGCGAAGGTA		
*PARKIN*	F:	TCCCAGTGGAGGTCGATTCT	218	XM_014858374.1
	R:	CCCTCCAGGTGTGTTCGTTT		
*MFN*	F:	AAGTGGCATTTTTCGGCAGG	217	XM_001495170.5
	R:	TCCATATGAAGGGCATGGGC		
*FIS*	F:	GGTGCGAAGCAAGTACAACG	118	XM_001504462.4
	R:	GTTGCCCACAGCCAGATAGA		
*GAPDH*	F:	GATGCCCCAATGTTTGTGA	250	XM_014866500.1
	R:	AAGCAGGGATGATGTTCTGG		
*TNF-α*	F:	ACAGAAAGCATGATCCGCGA	295	NM_013693.3
	R:	CTTGGTGGTTTGCTACGACG		
*IL-1β*	F:	TGCCACCTTTTGACAGTGATG	138	NM_008361.4
	R:	TGATGTGCTGCTGCGAGATT		
*IL-6*	F:	GAGGATACCACTCCCAACAGACC	141	NM_001314054.1
	R:	AAGTGCATCATCGTTGTTCATACA		
*GAPDH*	F:	TGCACCACCAACTGCTTAG	177	XM_017321385.1
	R:	GGATGCAGGGATGATGTTC		

*INFNG*—interferon gamma; *FOXP3*—forkhead box P3; *IL-10*—interleukin 10; *TGF beta*—tumor growth factor beta; *LC3*—microtubule-associated protein 1A/1B-light chain 3; *Beclin-1*; *LAMP-2*—Lysosome-associated membrane protein 2; *PINK-1*—PTEN-induced putative kinase 1; *PARKIN*—parkin ligase; *MFN*—mitofusin 1; *FIS*—mitochondrial fission 1 protein; *GAPDH*—glyceraldehyde 3-phosphate dehydrogenase; *TNF-α*—tumor necrosis factor alpha; *IL-1β*—interleukin 1 beta; *IL-6*—interleukin 6.
